# Application of Speckle Tracking Echocardiography for Evaluating Ventricular Function after Transcatheter Pulmonary Valve Replacement

**DOI:** 10.3390/diagnostics14010088

**Published:** 2023-12-30

**Authors:** Mengmeng Ji, Li Zhang, Lang Gao, Yixia Lin, Qing He, Mingxing Xie, Yuman Li

**Affiliations:** 1 Department of Ultrasound Medicine, Union Hospital, Tongji Medical College, Huazhong University of Science and Technology, Wuhan 430022, China; jimengmeng97@163.com (M.J.); zli429@hust.edu.cn (L.Z.); glcs@hust.edu.cn (L.G.); linyixia@hust.edu.cn (Y.L.); hqingedu@163.com (Q.H.); 2 Clinical Research Center for Medical Imaging in Hubei Province, Wuhan 430022, China; 3 Hubei Province Key Laboratory of Molecular Imaging, Wuhan 430022, China; 4 Shenzhen Huazhong University of Science and Technology Research Institute, Shenzhen 518057, China; 5 Tongji Medical College and Wuhan National Laboratory for Optoelectronics, Huazhong University of Science and Technology, Wuhan 430022, China

**Keywords:** speckle tracking echocardiography, transcatheter pulmonary valve replacement

## Abstract

Pulmonary regurgitation usually leads to right heart dilatation and eventually right heart dysfunction, which is associated with a poor prognosis. Transcatheter pulmonary valve replacement is a developing treatment for pulmonary valve dysfunction that can take the place of traditional surgery and make up for the shortcomings of a large injury. Echocardiography plays a significant role in assessing ventricular function; however, conventional echocardiographic parameters have several limitations. Speckle tracking echocardiography has been regarded as a more accurate tool for quantifying cardiac function than conventional echocardiography. Therefore, the aim of this review was to summarize the application of speckle tracking echocardiography for evaluating right and left ventricular functions in patients after transcatheter pulmonary valve replacement.

## 1. Introduction

Pulmonary regurgitation (PR) is a common complication in patients with various cardiovascular diseases, especially in repaired tetralogy of Fallot (TOF) patients, and is associated with a poor prognosis, including the dilatation of the right ventricular outflow tract (RVOT), right ventricular (RV) dysfunction, tricuspid regurgitation, arrhythmia, sudden death, and other conditions [1,2,3,4,5,6]. An earlier study found that approximately 40% of these patients required a pulmonary valve replacement (PVR) thirty-five years after a repaired TOF [1]. Long-term PR after a repaired TOF is an important determinant of postoperative cardiovascular death, and therefore prompt interventions in PR can alter the natural course of this disease. PVR is recommended by the American College of Cardiology, the American Heart Association, and the European Society of Cardiology for symptomatic patients with moderate or severe PRs after TOF repair [7,8].

The lifespan of a prosthetic pulmonary valve that has been surgically replaced, however, is constrained, and patients frequently require several PVR procedures over their lives, which increases complications and decreases survival rates [9,10]. The first successful transcatheter pulmonary valve replacement (TPVR) was reported by Bonhoeffer et al. in 2000 [11]. The Melody (Medtronic, Minneapolis, MN, USA) and the SAPIEN platforms (Edwards Lifesciences, Irvine, CA, USA) are the currently widely used balloon-expandable systems for TPVR. TPVR is an option to enhance cardiac function and extend life in these patients, and can address the drawbacks of surgical PVR, including significant injury, high risk, and multiple complications. For individuals who are high risk or have surgical contraindications, TPVR offers a procedure with notable success and less trauma, and it has been considered as a substitute for PVR [9,12]. In addition, TPVR can improve the symptoms of patients, reverse ventricular remodeling, improve cardiac function, and postpone and reduce the times of open-heart operations. Therefore, it can extend the life of conduits that are surgically implanted from the RV to the pulmonary artery (PA) and potentially extend the life expectancy of these patients [9,10]. Although studies have shown that PVR and TPVR have the same perioperative mortality rates, mid-term mortality, and reintervention rate, TPVR has a lower incidence of perioperative complications and shorter hospital stay [13,14]. The 2020 European Society of Cardiology guideline for the management of congenital heart disease indicates that TPVR is preferred in patients with a suitable anatomy [15]. Currently, there are more than twenty-thousand TPVR applications worldwide [16]. The accurate measurement of the changes in ventricle function during a postoperative follow-up session is essential for developing treatment strategies and enhancing prognoses since ventricle function after TPVR is closely associated with outcomes. The majority of previous studies have demonstrated that the evaluation of left ventricular (LV) function is the core part of the clinical management of cardiovascular disease and is of great significance to guide treatment and predict clinical outcomes [17,18,19,20]. Likewise, an increasing body of research has shown that the early detection of RV dysfunction is able to predict the adverse outcomes of numerous cardiovascular diseases [21,22,23,24]. The assessment of biventricular function should be considered in clinical practice since the left and right ventricles form a unit that is closely connected by superficial myofibers, the interventricular septum (IVS), and pericardium [25,26]. Cardiac magnetic resonance imaging (CMR) is regarded as the “gold standard” of ventricular function evaluations; however, its widespread application is hampered by the inherent disadvantages, including high cost, longer scan time, and contraindications of metal implants [22,27,28,29]. In clinical practice, echocardiography is frequently utilized to evaluate cardiac function due to its availability, practicality, and lower cost. As a result, echocardiography is usually considered as the first choice for patients after TPVR during the postoperative follow-up period [30,31]. However, conventional echocardiography has certain limitations for evaluating RV function due to its complex three-dimensional geometry. Speckle tracking echocardiography (STE), which has recently come into existence, offers a novel approach for precisely determining biventricular performance. STE can overcome the defects of angle dependency of tissue Doppler imaging (TDI) and detect subclinical ventricular dysfunction in the early stage, which proves its superiority over traditional echocardiography parameters [17,32,33,34,35]. Consequently, the aim of this review is to summarize the application of STE for assessing the biventricular function in patients after TPVR.

## 2. The Influence of Severe PR on Ventricular Structure and Function

The normal flow from the right atrium and abnormal flow from PR flow into the right ventricle together during diastole in patients with PRs, increasing the RV volume load and perhaps contributing to RV dilation. In the meantime, the blood flow in PA also increases. Both the RV pressure load and the ventricular wall tension increase during an RV ejection when PR is combined with pulmonary hypertension, which can aggravate RV dilation and result in RV hypertrophy.

The RV systolic function is initially preserved in patients with chronic PRs. This stage can last for several years, and lots of patients remain relatively free of symptoms. However, with the progress of PR, several studies show a close relationship between the severity of PR and RV end-diastolic volume [36]. Chronic severe PR finally leads to a decrease in RV function, systolic ventricular deterioration, and the establishment of myocardial fibrosis, which can convert RV dysfunction into something irreversible. On account of the close relationship between the right and left ventricles, RV contractile dysfunction further results in LV systolic dysfunction [37,38]. Davlouros et al. indicated that LV contractile dysfunction was related to RV dysfunction in patients with a repaired TOF, which suggested the existence of an unfavorable ventricular-ventricular interaction [39].

## 3. Two-Dimensional Echocardiography and Tissue Doppler Imaging

The altered systolic and diastolic parameters of two-dimensional (2D) echocardiography and TDI suggest that long-term and severe PRs lead to a number of adverse changes in myocardial function (Table 1). PR usually contributes to a reduced RV fractional area change (RVFAC), tricuspid annular plane systolic excursion (TAPSE), RV TDI S′, and increased RV end-diastolic diameter (RVEDD). Long-term RV dysfunction frequently accompanies LV dysfunction in patients with a PR, which is usually demonstrated as a reduced LV ejection fraction (LVEF) and LV TDI S′, and increased LV end-diastolic diameter (LVEDD) [30,40,41].

TDI allows for a more accurate time definition and reflects myocardial velocities, strain, and strain rate, which can provide significant information about cardiac function. Strain measurements based on the myocardial velocity gradient can estimate the strain rate, and therefore strain can be calculated as the temporal integral of the strain rate. Strain and strain rate are measured in the apical view with a favorable myocardial movement along the ultrasound beam [42]. TDI has high frame rates, which is helpful for detecting rapid changes in deformations and tracking the deformation in patients with rapid heart rates [43]. In addition, the advantages of TDI also include simplicity, high spatial resolution, and fast sampling, so it can be widely used in clinical and scientific research [44,45,46,47,48,49,50]. However, TDI also has several limitations that involve angle dependency, influenced by the preload and afterload, a low signal-to-noise ratio, and the evaluation of motion information confined to myocardial segments that move along the direction of the ultrasound beam [51]. Reduced myocardial function has been demonstrated by a lower myocardial tissue velocity, strain, and strain rate in patients with severe PRs [52].

## 4. Speckle Tracking Echocardiography

### 4.1. General

STE is an advanced quantitative echocardiographic technique that assesses myocardial function by analyzing the motion of speckles that are created through the interaction of an ultrasound with myocardial fibers [53,54]. The endocardium and epicardium are delineated first during the STE analysis, and then the myocardial deformation is evaluated through tracking the motion of speckles during systole and diastole [55]. Myocardial strain measured by STE includes two components of myocardial movement velocity and direction to quantitatively analyze the contractile functions of each segment, which can detect cardiac dysfunction more precisely and sensitively [17]. Myocardial strain, which comprises the spatial components of global and regional myocardial strains in longitudinal, circumferential, and radial directions, is defined as the percentage change in the length of a myocardial segment relative to its initial length at the end diastole [56]. The strain rate is typically used to measure the velocity of cardiac deformation [55,57]. Due to the fact that STE is based on standard 2D or 3D echocardiography methods rather than Doppler echocardiography, and has the main benefit of angle independence, it has been utilized to assess myocardial function more frequently in recent years. Also, strain rate measurements show a reduced load dependence. In addition, they have low interobserver variabilities and are not susceptible to respiratory movements or heartbeat [34,49,54,58,59,60,61,62].

### 4.2. Strain Parameters

The myocardial shortening of the ventricular length from the base to the apex is referred to as the longitudinal strain (LS). The radial strain (RS) reflects a radial myocardial deformation in the short-axis view during systole. In the same manner as the RS, the circumferential strain (CS) is a deformation measured along the circular perimeter; however, the CS curve is often negative [63].

**Figure 1:**
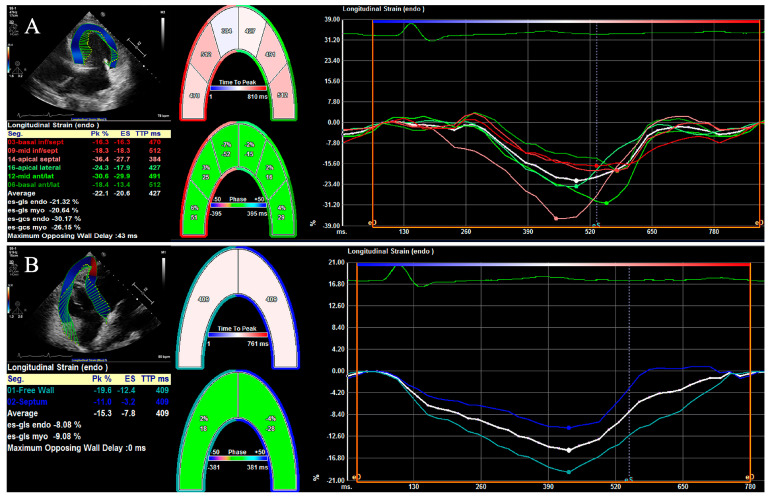
Longitudinal strains of left and right ventricles using two-dimensional speckle tracking echocardiography. (**A**) Longitudinal strains of left ventricle for each segment and left ventricular global longitudinal strain. (**B**) Longitudinal strain of right ventricular free wall and septum.

LV shortens longitudinally and circumferentially, but thickens radially during systole [64]. The LV global longitudinal strain (LVGLS), which overcomes the impact of regional noise and is less impacted by geometrical confounders [65], is obtained from the average of LS values from all LV segments in the standard, apical, long, two-chamber and four-chamber views. Previous investigations have demonstrated that the LVGLS can provide superior diagnostic and prognostic information over conventional echocardiography parameters, LV global circumferential strain (LVGCS), and LV global radial strain (LVGRS) [66], and, therefore, the LVGLS is considered as a notably robust and sensitive systolic function indicator [64,67,68,69,70,71]. The 2D-STE analysis for the left ventricle is shown in Figure 1A.

The right ventricle is composed of longitudinal inner and outer myocardium fibers and lacks circumferential middle myocardium fibers. Hence, the RV longitudinal strain (RVLS) contributes most to the overall RV contraction. An abnormal RVLS, which usually appears in subclinical RV systolic dysfunction, can provide prior sensitivity over the RV circumferential strain (RVCS) and RV radial strain (RVRS) and contribute to offering critical prognostic information in various cardiovascular diseases [22,34,72]. The RV global longitudinal strain (RVGLS) is calculated as the average of the six RV segments, while RV free wall longitudinal strain (RVFWLS) is calculated as the average of three segments, involving basal, middle, and apical segments, of the RV free wall [35,73,74,75]. The 2D-STE analysis for the right ventricle is shown in Figure 1B. Recently, the published guidelines of the ASE and the European Association of Cardiovascular Imaging (EACVI) recommended a normal value only for the RVFWLS, for the reason that the RVGLS could be influenced by the LV systolic function [76].

### 4.3. Two-Dimensional Speckle Tracking Echocardiography

The 2D-STE method based on 2D gray-scale imaging at a high frame rate can identify a myocardial deformation along the direction of the ultrasound beam, both in the circumferential and radial directions, with the advantages of easy operation and high repeatability [77]. However, 2D-STE has several intrinsic limitations. At first, a good image quality is necessary for an accurate strain analysis, which facilitates the precise definition and tracing of the endocardial border. Furthermore, 2D-STE based on 2D imaging is also limited by the out-of-plane motion of speckles because cardiac motion is 3D in nature [78,79,80,81].

### 4.4. Three-Dimensional Speckle Tracking Echocardiography

**Figure 2:**
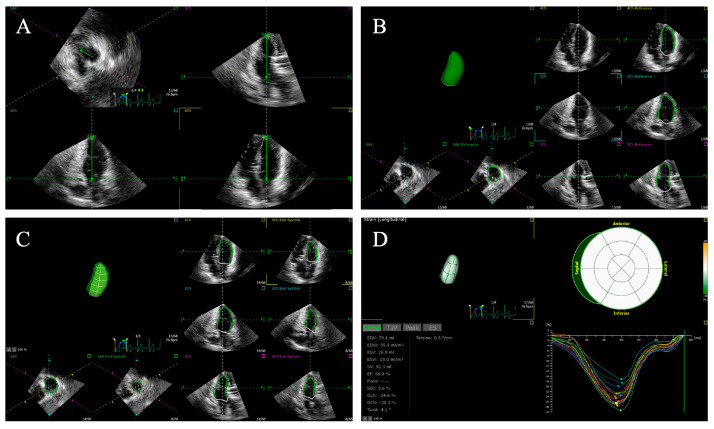
Left ventricular global longitudinal strain using three-dimensional speckle tracking echocardiography. (**A**) Setting reference points; (**B**,**C**) left ventricular endocardial border tracking; (**D**) left ventricular global longitudinal strain is automatically generated.

**Figure 3:**
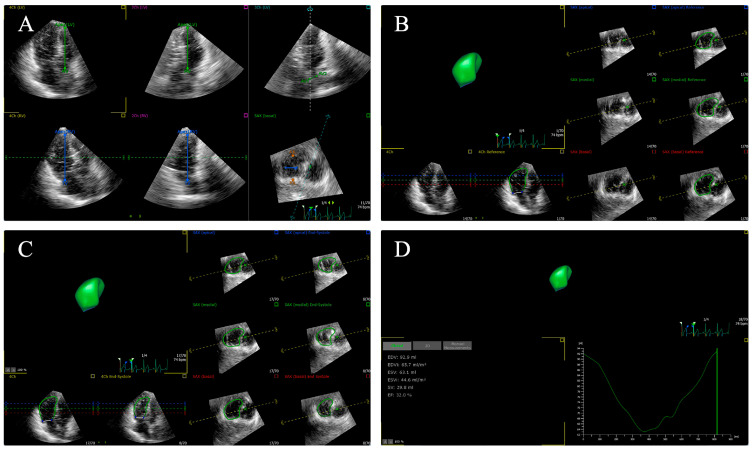
Longitudinal strain of right ventricular free wall and septum using three-dimensional speckle tracking echocardiography. (**A**) Setting reference points; (**B**,**C**) right ventricular endocardial border tracking; (**D**) longitudinal strain of right ventricular free wall and septum are automatically generated.

Recently, three-dimensional speckle-tracking echocardiography (3D-STE) that can track myocardial movement in three-dimensional volume was introduced as a novel echocardiographic technique. The 3D-STE method based on real-time full-volume scanning is free of geometric assumptions and overcomes the shortcomings of the out-of-plane motion of speckles in the 2D-STE method [82,83]. Although the 2D-STE method for ventricular strain is a widely accepted echocardiographic technology for evaluating LV and RV functions and detecting subclinical ventricular abnormalities, 3D-STE allows a more detailed and realistic evaluation in a shorter period of time, which involves the simultaneous assessment of volume, strain, and rotation of the ventricle [84]. The 3D-STE method can calculate strain values in all directions at the same time, which can quantify myocardial deformation more accurately and evaluate cardiac function more objectively and comprehensively [34,85,86,87,88,89]. Additionally, compared with 2D-STE, 3D-STE has been demonstrated to be better related to CMR, which is usually regarded as the standard non-invasive technique for evaluating myocardial strain [83,90,91]. However, the clinical application of 3D-STE is limited by its low temporal and spatial resolution and dependence on image quality [55,92,93]. The 3D-STE analyses for the left and right ventricles are shown in Figure 2 and Figure 3, respectively.

## 5. The Application of Speckle Tracking Echocardiography in Patients after TPVR

### 5.1. The Global Longitudinal Strain and Strain Rate of the Right Ventricle

The RV volume overload caused by PR can be relieved by TPVR, which can improve cardiac function before discharge. The current findings regarding RVLS and the longitudinal strain rate of RV (RVLSR) derived from 2D-STE after TPVR are depicted in Table 2. Moiduddin et al. [52] used 2D-STE to study four patients with simple PRs and six patients with PR and pulmonary stenosis (PS) after repaired TOFs, and they found that RVFWLS increased and RVEDD significantly decreased, while other conventional echocardiographic parameters of RV function did not differ before being discharged compared with the baseline values. This study demonstrated that RV overload could be alleviated by TPVR, which led to an improved RV function.

Chowdhury et al. [94] carried out a study by 2D-STE in twenty-four patients with PRs as the indication of TPVRs: twenty-two patients with severe PRs, one patient with a mild PR, and one patient with a moderate PR. Additionally, most of the patients were combined with PSs of varying degrees. RVGLS, RV global longitudinal strain rate (RVGLSR), RVFWLS, and RV free wall longitudinal strain rate (RVFWLSR) showed varying degrees of improvements one month after TPVR. However, RVFAC, TAPSE, and other conventional echocardiographic parameters of RV were not different after TPVR compared with the preoperative values. Chowdhury et al. also regarded the ventilatory efficiency derived from exercise testing, the minute ventilation [$V_{E}$]/carbon dioxide production [$V_{{CO}_{2}}$] slope, as the predictive indicator of mortality. Statistically significant improvements in RVGLS, RVGLSR, RVFWLS, RVFWLSR, the RV global early diastolic global longitudinal strain rate (RVGLSRe), and RV free-wall early diastolic global longitudinal strain rate (RVFWLSRe) were detected at the 6-month follow-up session, while no significant changes were noted in the traditional echocardiographic parameters of RV systolic or diastolic functions. The change in $V_{E}/V_{{CO}_{2}}$ was only significantly related to the changes in RVGLS, RVGLSRe, RVFWLS, and RVFWLSRe. The significant relationship with percentage change in $V_{E}/V_{{CO}_{2}}$ was only noted in the percentage changes in RVGLSRe, RVFWLSRe, and tricuspid valve inflow Doppler A velocity on multiple variable regressions. In addition, the patients with lower RVGLS and RVFWLS values before TPVR showed the most obvious improvement in $V_{E}/V_{{CO}_{2}}$ postoperatively. And only RVGLS and RVFWLS before TPVR were closely correlated with the percentage change in $V_{E}/V_{{CO}_{2}}$ after TPVR in the univariate and multivariate analyses. The improvements in RVGLS, RVGLSRe, RVFWLS, and RVFWLSRe indicated that STE could detect subclinical dysfunctions before the irreversible deterioration of RV function early; additionally, abnormal RVGLS and RVFWLS before TPVR contributed to predicting the prognosis after TPVR.

In another study by Moiduddin et al. [40], 2D-STE was applied to investigate the RVGLS in nine patients with a variant of TOF and one patient with complicated LV outflow tract obstruction requiring a Ross and RV pulmonary atresia conduit. The indications for TPVR were PR in six cases, PS in two cases, and PR combined with PS in two cases. The RV late diastolic global longitudinal strain rate (RVGLSRa) immediately significantly improved before discharge after TPVR compared with these parameters before TPVR, while RVGLS and RVGLSR remained stable three months after TPVR compared with discharge. And, at six months after TPVR, the RV systolic global longitudinal strain rates (RVGLSRs) were significantly decreased compared with those before discharge. As the degree of PR decreased after TPVR, the RV stroke output decreased rapidly, and therefore RVGLS and RVGLSR dramatically decreased in patients with TPVRs due to PR [95]. And, with the ventricular remodeling and recovery of cardiac function, RVGLS and RVGLSR returned to baseline levels at the 6-month follow-up session. On the contrary, in patients with TPVRs due to PS, RV stiffness was reduced due to decreased PA resistance and RV end-diastolic pressure after TPVR, resulting in significant improvements in RVGLS and RVGLSR. However, possibly because RV remodeling and function were affected by childhood hypoxia and multiple previous operations, RVGLS and RVGLSR returned to the baseline levels again after six months of follow-up treatment. The changes in the strain parameters in the PR group combined with the PS group were between those in the PR and PS groups.

Chowdhury et al. [96] also conducted another study by 2D-STE, and they demonstrated that the traditional echocardiographic parameters of RV function and RVGLSRe were not obviously changed, while RVGLS and RVGLSR improved at six months after TPVR. The tricuspid TDI E’ velocity increased rapidly after TPVR, returned to the preoperative level one month after surgery, and remained relatively stable at six months after TPVR.

**Table 2:** RV, LV, and IVS longitudinal strains and strain rates after TPVR.

Study	N	Age (Y)	Diagnosis	TPVR Indication	Follow Up	RVLS (%)	RVLSR (s^−1^)	LVLS (%)	LVLSR (s^−1^)	IVSLS (%)	IVSLSR (s^−1^)
Moiduddin et al. [52]	10	15.56 ± 2.22	TOF: 7 Other: 3	PR: 4 PR+PS: 6	pre-discharge	RVFWLS: −23.4 ± 6.2	−2.1 ± 0.7	−20.0 ± 11.2	−1.5 ± 0.5	−15.6 ± 6.7	−1.13 ± 0.5
Chowdhury et al. [94]	24	32.3 ± 17.0	TOF: 12 Other: 12	PR: 7 PR+PS: 17	1 month	RVGLS: −17.8 ± 0.6 RVFWLS: −19.1 ± 4.8	RVGLSR: −1.03 ± 0.05 RVGLSRe: 1.12± 0.09 RVFWLSR: −1.11 ± 0.30 RVFWLSR: 1.27 ± 0.61	LVGLS: −18.0 ± 1.1	LVGLSR: −1.11 ± 0.08 LVGLSRe: 1.30 ± 1.10	−15.9 ± 2.9	−0.93 ± 0.27
					6-month	RVGLS: −19.6 ± 0.9 RVFWLS: −21.9 ± 6.2	RVGLSR: −1.16 ± 0.08 RVGLSRe: 1.31± 0.10 RVFWLSR: −1.31 ± 0.68 RVFWLSRe: 1.43 ± 0.64	LVGLS: −18.2 ± 0.9	LVGLSR: −1.06 ± 0.15 LVGLSRe: 1.32 ± 0.09	−17.8 ± 5.4	−1.05 ± 0.42
Chowdhury et al. [96]	24	32.3 ± 17.0	TOF: 12 Other: 12	PR: 7 PR+PS: 17	6-month	−19.6	−1.16	−18.2	-	-	-
Moiduddin et al. [40]	10	24.4 ± 7.6	TOF: 9 Other: 1	PR: 6 PS: 2 PR+PS: 2	pre-discharge	RVGLS: −17.13 ± 2.71 basal: −19.71 ± 5.18 mid: −15.96 ± 5.45 apical: −16.96 ± 6.65	RVGLSRs: −0.97 ± 0.22 RVGLSRe: 1.05 ± 0.32 RVGLSRa: 0.61 ± 0.14	LVGLS: −19.23 ± 1.49 basal: −22.87 ± 5.09 mid: −18.64 ± 3.68 apical: −19.64 ± 4.69	LVGLSRs: −1.06 ± 0.10 LVGLSRe: 1.23 ± 0.32 LVGLSRa: 0.63 ± 0.21	basal: −17.83 ± 3.73 mid: −20.55 ± 2.19 apical: −20.27 ± 4.27	-
					3-month	RVGLS: −16.96 ± 5.17 basal: −20.27 ± 5.60 mid: −15.55 ± 7.57 apical: −16.22 ± 9.21	RVGLSRs: −0.87 ± 0.28 RVGLSRe: 1.03 ± 0.36 RVGLSRa: 0.49 ± 0.29	LVGLS: −14.72 ± 3.62 basal: −19.67 ± 10.46 mid: −12.29 ± 5.64 apical: −15.20 ± 6.89	LVGLSRs: −0.84 ± 0.16 LVGLSRe: 1.11 ± 0.44 LVGLSRa: 0.29 ± 0.28	basal: −15.41 ± 3.16 mid: −17.77 ± 3.47 apical: −15.71 ± 3.99	-
					6-month	RVGLS: −16.95 ± 4.20 basal: −20.97 ± 7.68 mid: −15.04 ± 7.02 apical: −16.49 ± 5.88	RVGLSRs: −0.83 ± 0.22 RVGLSRe: 1.08 ± 0.28 RVGLSRa: 0.52 ± 0.21	LVGLS: −17.18 ± 3.08 basal: −24.72 ± 9.31 mid: −14.00 ± 6.60 apical: −17.10 ± 3.92	LVGLSRs: −0.94 ± 0.23 LVGLSRe: 1.23 ± 0.28 LVGLSRa: 0.50 ± 0.15	basal: −16.09 ± 5.47 mid: −18.66 ± 3.33 apical: −18.23 ± 6.20	-
Hasan et al. [41]	20	18	TOF:13 Other: 7	PR+ obstructed RVOT conduit: 9 obstructed RVOT conduit: 11	6-month	RVGLS: −17.0 (−12, −22) ^a^; −18.8 (−13, −24) ^b^ RV lateral wall strain: −18.3 (−6.8, −28.3) ^a^; −18.6 (−11, −32) ^b^	-	LVGLS: −18.6 (−14.6, −22.0) ^a^; −20.8 (−15.4, −23.0) ^b^ LV lateral wall strain: −20.3 (−16.7, −25) ^a^; −22.3 (−18.5,1 −27) ^b^	-	−16.6 (−11, −21) ^a^; −17.2 (−12.3, −23.0) ^b^	-

Values are mean ± standard deviation, median, or median (minimum, maximum). ^a^ at rest; ^b^ during exercise. TPVR = transcatheter pulmonary valve replacement; RVLS = right ventricle longitudinal strain; RVLSR = right ventricle longitudinal strain rate; LVLS = left ventricle interventricular septum; LVLSR = left ventricle longitudinal strain rate; IVSLS = interventricular septum longitudinal strain; IVSLSR = interventricular septum longitudinal strain rate; TOF = tetralogy of Fallot; PR = pulmonary regurgitation; PS = pulmonary stenosis; RVFWLS = right ventricular free wall longitudinal strain; RVGLS = right ventricular global longitudinal strain; RVGLSR = right ventricular global longitudinal strain rate; RVGLSRe = right ventricular early diastolic global longitudinal strain rate; RVFWLSR = right ventricular free wall longitudinal strain rate; RVFWLSRe = right ventricular early diastolic free wall longitudinal strain rate; LVGLS = left ventricular global longitudinal strain; LVGLSR = left ventricular global longitudinal strain rate; LVGLSRe = left ventricular early diastolic global longitudinal strain rate; RVGLSRs = right ventricular systolic global longitudinal strain rate; RVGLSRa = right ventricular late diastolic global longitudinal strain rate; LVGLSRs = left ventricular systolic global longitudinal strain rate; LVGLSRa = left ventricular late diastolic global longitudinal strain rate; and RVOT = right ventricular outflow tract.

Hasan et al. [41] applied 2D-STE to evaluate ventricular function in twenty patients with an obstructed right ventricular outflow tract (RVOT) conduit during exercise and at rest before and six months after TPVR. RVGLS severely decreased at baseline rest compared with the controls, who significantly improved at rest six months after TPVR. In addition, RVGLS during exercise modestly increased compared with the rest state after TPVR. The increases in RVFWLS and RVGLS during exercise and at rest after TPVR indicated an improvement in the RV systolic function. This study also showed that higher RV strain values before the intervention were associated with higher RV strain values after TPVR, suggesting that the better RV function before TPVR could be related to the benefit of removing obstructions to the RV function after TPVR, which could help determine the timing of early interventions in patients with RVOT obstructions.

The improvements in ventricular strain parameters at six months after TPVR were earlier than that of conventional echocardiographic parameters, suggesting that the strain parameters derived from STE were more sensitive in assessing the improvement in cardiac function after TPVR. The abnormal RVGLS before TPVR was a predictor of improved prognosis after TPVR. The application of STE to evaluate RV function before and after TPVRs has potential clinical value. At the same time, STE could contribute to determining the optimal timing of interventions for patients with RVOT obstructions. Therefore, the STE strain analysis is of great significance for patients who plan to undergo TPVR.

### 5.2. The Global Longitudinal Strain and Strain Rate of the Left Ventricle

As the biventricle is a tightly connected unit, RV dysfunction can affect LV function. Therefore, the evaluation of LV performance also plays a significant role in patients after TPVR. The current findings regarding LVGLS and the global longitudinal strain rate of LV (LVGLSR) derived from 2D-STE after TPVR are depicted in Table 2.

The STE analysis is more sensitive to detect the improvement of myocardial systolic function when there is no significant change in the myocardial contractile velocity evaluated by TDI. The study [94] applied by 2D-STE demonstrated that the LVGLS and LVGLSR improved one month after TPVR. However, other conventional echocardiographic parameters of LV function were not different after TPVR compared with the preoperative values. They conducted a further study [96] on the sample and found that the traditional echocardiographic parameters of LV function, LVGLSR, and LVGLSRe were not obviously changed; however, LVGLS improved at six months after TPVR. In addition, the TDI parameters of cardiac systolic function, including tricuspid annulus E’ and lateral mitral annulus E/E’, presented no significant differences. Therefore, they demonstrated that TDI was more load dependent than STE in assessing the LV systolic function and volume-loaded right ventricle [97,98,99,100]. The improvements in the strain and strain rate of the left and right ventricles between pre-operation and one month after TPVR can be explained by the autoregulatory mechanisms accompanying loading changes [101].

In the abovementioned study by Moiduddin et al. [40], they also confirmed that LVGLS immediately significantly improved before discharge after TPVR compared with these parameters before TPVR. However, compared with discharge, there were significant decreases in the LVGLS, LV systolic global longitudinal strain rate (LVGLSRs), and LV late diastolic global longitudinal strain rate (LVGLSRa) at three and six months after TPVR.

### 5.3. The Regional Longitudinal Strain Values of the Left and Right Ventricles

RVGLS is defined as the average of the six RV segments; in addition, the LV lateral wall and IVS can also be divided into three segments: basal, middle, and apical. Therefore, several studies have elaborated the changes in the regional longitudinal strain of the left and right ventricles in patients after TPVR, and the current findings are shown in Table 2.

The study by Moiduddin et al. [52] showed that the IVS longitudinal strain (IVSLS) and IVS longitudinal strain rate (IVSLSR) increased, while the LVGLS and other conventional echocardiographic parameters of the LV did not obviously change before being discharged after TPVR. Additionally, the increases in IVSLS and IVSLSR indicated that the RV function could be affected by IVS, which was in accord with the viewpoints proposed by several researchers that IVS had a compensatory impact on the impaired RV free wall function [102].

In the abovementioned study by Moiduddin et al. [40], they also investigate regional biventricular longitudinal strains in nine patients. And there were significant decreases in the longitudinal strains of each segment of IVS and the middle segment of the left ventricle (LV-mid) at three months after TPVR compared with discharge, while the longitudinal strain and strain rate of RV for each segment remained stable. They also found that the longitudinal strains of IVS-mid, LV-mid, and RV-basal segments were significantly decreased at six months after TPVRs compared with those before discharge. In this study, the longitudinal strain of each segment of IVS improved evidently, while the regional RV function did not improve, and the longitudinal strain of RV-basal segment was significantly reduced in the whole cohort. This indicated that cardiac function could be improved by an interventricular interaction after TPVR relieved RV volume overload, and the rapid improvement of the IVS strain could compensate for the impaired function of the RV-basal segment. In addition, the strain assessment was closely related to ventricular load status and geometry, and therefore the use of strain parameters to evaluate ventricular function must be interpreted in the context of ventricular load status and geometry [103,104,105].

Hasan et al. [41] also showed the regional longitudinal strains of left and right ventriclels at baseline and six months after TPVR. They showed that the RV lateral wall and IVS median peak longitudinal strain severely decreased, and the IVS longitudinal strain moderately decreased at baseline rest compared with the controls. The RV lateral wall, LV lateral wall, and IVS median peak longitudinal strain significantly improved during rest six months after TPVR. The RV lateral wall and IVS median peak longitudinal strain significantly improved during exercise after TPVR. In addition, the IVS and LV lateral wall median peak longitudinal strains during exercise were higher compared with the corresponding strain values at rest before TPVR, while the LV lateral wall median peak longitudinal strain during exercise modestly increased compared with the rest state after TPVR.

## 6. Conclusions

TPVR can overcome the shortcomings of traditional surgical PVR, reduce the risk of sudden death, and improve the quality of life for patients with PRs. STE is able to sensitively and accurately assess the changes in ventricular function during follow-up sessions, contributing to creating clinical treatment plans and improving outcomes. At present, most studies focus on the application of 2D-STE to evaluate the changes in ventricular function within six months after TPVR, while 3D-STE, as a new technology to evaluate ventricular function, can overcome the limitations of 2D-STE. Therefore, 3D-STE should be considered as a vital tool during follow-up treatment after TPVR in future studies so that the changes in the biventricular function in patients after TPVR can be more accurately evaluated and a reference for clinical decision making can be provided.

## Figures and Tables

**Table 1:** Changes in echocardiographic parameters in patients with long-term and severe pulmonary regurgitations as measured by two-dimensional echocardiography and tissue Doppler imaging.

Two-Dimensional Echocardiographic Parameters	Effects of Long-Term and Severe PRs	TDI Parameters	Effects of Long-Term and Severe PRs
RV Parameters			
RVFAC	↓	TDI S′	↓
TAPSE	↓	TDI RV strain	↓
RVEDD	↑	TDI RV strain rate	↓
LV Parameters			
LVEF	↓	TDI S′	↓
LVEDD	↑	TDI LV strain	↓
		TDI LV strain rate	↓

2D = two dimensional; PR = pulmonary regurgitation; TDI = tissue Doppler imaging; RV= right ventricle; RVFAC = right ventricular fractional area change; TAPSE = tricuspid annular plane systolic excursion; RVEDD = right ventricular end-diastolic diameter; LV = left ventricle; LVEF = left ventricular ejection fraction; LVEDD = left ventricular end-diastolic diameter

## Data Availability

Not applicable.
